# Soil micronutrients linked to human health in India

**DOI:** 10.1038/s41598-023-39084-8

**Published:** 2023-08-21

**Authors:** Claire M. Morton, Hemant Pullabhotla, Leah Bevis, David B. Lobell

**Affiliations:** 1 Mathematical and Computational Science Program, Stanford University, Stanford, USA; 2 Department of Economics, Deakin University, Burwood, Australia; 3 Department of Agricultural, Environmental and Development Economics, Ohio State University, Columbus, USA; 4 Department of Earth System Science and Center on Food Security and the Environment, Stanford University, Stanford, USA

**Keywords:** Risk factors, Socioeconomic scenarios

## Abstract

Trace soil minerals are a critical determinant of both crop productivity and the mineral concentration of crops, therefore potentially impacting the nutritional status of human populations relying on those crops. We link health data from nearly 0.3 million children and one million adult women across India with over 27 million soil tests drawn from a nationwide soil health program. We find that soil zinc availability is positively associated with children’s linear height growth, and soil iron availability is positively associated with hemoglobin levels. The link between soil zinc and childhood stunting is particularly robust—a one standard deviation increase in satisfactory soil zinc tests is associated with approximately 11 fewer children stunted per 1000. We also find that this zinc-stunting relationship is strongest in wealthier households. Our results suggest that soil mineral availability impacts human nutritional status and health in at least some areas of India, and that agronomic fortification may be a beneficial intervention.

## Introduction

Over two billion people globally are micronutrient deficient^1^. Micronutrient deficiency has been shown to impact learning, IQ, motor skills, and immune system function, and for children who were micronutrient-deficient, it has lasting effects through adulthood^2,3^. Zinc deficiency, in particular, was estimated to have caused 116,000 deaths in children under five years old globally in 2011^4^. Additionally, 175 million more people may be zinc deficient by 2050 due to globally rising $\hbox {CO}_2$ levels^5^.

In the developing world, a large share of the population is possibly at a greater risk for mineral deficiencies because they consume crops grown on soils that are low in bioavailable mineral concentration. Low soil mineral availability causes the edible portion of many cereals, legumes, and vegetables to be low in mineral concentration^6^, and mineral-enriched fertilizers could potentially rectify this problem^7^. Several countries (Finland, China, and Turkey) have therefore employed agronomic fortification—enriching fertilizers or irrigation water with trace minerals such as selenium, iodine, and zinc—in what appear to have been successful efforts to raise crop mineral concentration and domestic human mineral status^8,9^.

While there is a dearth of strictly experimental evidence illustrating the human nutrition impacts of agronomic fortification, a number of studies quantify spatial correlations between soil and human mineral deficiencies. Much of this work has focused on Africa. For instance, serum zinc levels were correlated with weight-for-height Z-scores among children in Ethiopia^10,11^, and selenium-rich soils were associated with selenium status of nearby cereal samples, and with adequate selenium status for children in Malawi^12–14^. In an Africa-wide study, children’s health outcomes were also strongly correlated with soil densities of zinc, copper, and manganese^15^. However, estimates from these and other studies have found that applying mineral-enriched fertilizers was not as cost-efficient as other health interventions^15,16^.

Few studies have examined the link between soil quality and human health outside Africa. One exception is Bevis et al. (2023), who find that child stunting is correlated with soil zinc availability in Nepal’s Tarai region, even when controlling for a wide variety of soil, demographic, and environmental characteristics^17^. Ultimately, more research in this area is necessary to determine whether the relationship between soil nutrient quality and children’s health outcomes extends to other regions of the world.

To date, no large-scale studies have examined the association between children’s nutritional status or health outcomes and soil mineral availability in India^18^. Yet India contains roughly one-third of the global population suffering from micronutrient deficiency^19^. The rate of child stunting in India is about 35%^20^, and malnutrition was the leading risk factor for loss of Disability-Adjusted Life-Years in 2016, causing an estimated 0.5% of all deaths in India^21^. Additionally, almost 138 million people in India—10% of the country’s population—are rural residents living below the poverty line^22,23^. Many of these people are farmers who own small amounts of land and rely on their own production for food, particularly for staple cereals^24,25^. Over 35% of soils in India are estimated to be deficient in zinc, and about 11% of soils in India are estimated to be deficient in iron^8,26^.

**Figure 1:**
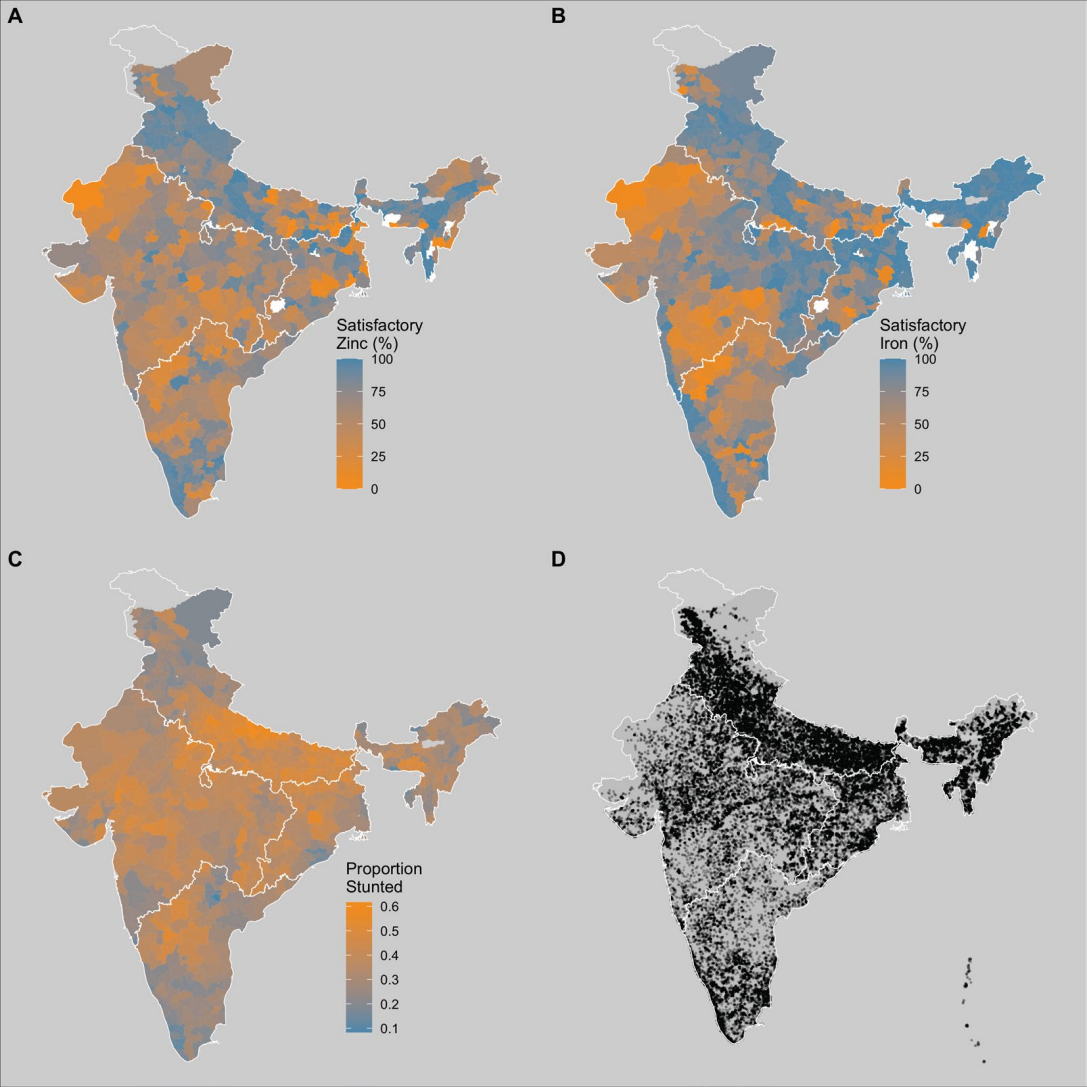
Soil and health data summary, mapping at a district level: The proportion of soil samples deemed to be satisfactory in (**A**) zinc availability and (**B**) iron availability, according to government standards, and (**C**) the proportion of children determined to be stunted based on WHO height-for-age z-score. Also mapping: (**D**) NFHS cluster locations. Regions are outlined in white. Maps were created in RStudio version 2021.09.0.

This study evaluates the relationships between several child and adult health outcomes and soil mineral availability in India (Fig. 1C,D). We examine the association between soil zinc availability and child stunting or women’s height because human Zn deficiency is generally found to inhibit linear growth^27–32^. We also examine the association between soil iron availability and child or women’s anemia because iron deficiency is the primary cause of anemia in India and more generally^33,34^. We utilize over 27 million soil tests conducted in recent years by the Indian government (Fig. 1A,B). This is the densest soil quality data ever used in a study of this type by over two orders of magnitude^15^. We use these data to construct exposure to soil mineral availability at the district level encompassing the whole of the country.

## Results

### Main results

Our main results model each health outcome of interest on trace soil mineral availability using linear regression specifications. We measure exposure to soil mineral availability as the percent of soil tests in an individual’s district of residence deemed satisfactory for the given trace mineral. In addition to the soil variables, our linear models include controls for numerous individual and household characteristics and region or state fixed-effects to account for unobserved spatial variables (see "Methods" Section). This cross-sectional methodology is similar to methods employed by related studies^17^. However, instead of individual location-specific soil mineral availability, we use the district-level measure of soil mineral availability.

The health outcomes in our analysis consist of anthropometric measures and hemoglobin levels for children, and height and hemoglobin for adult women in the sample. Past studies suggest that soil zinc may impact children’s nutritional outcomes in some settings^10,11,17^. But evidence is limited on the link between soil zinc and long-term heights attained by adults. We also explore the link between soil iron availability and hemoglobin levels. To the best of our knowledge, no previous study has tested this relationship.

**Figure 2:**
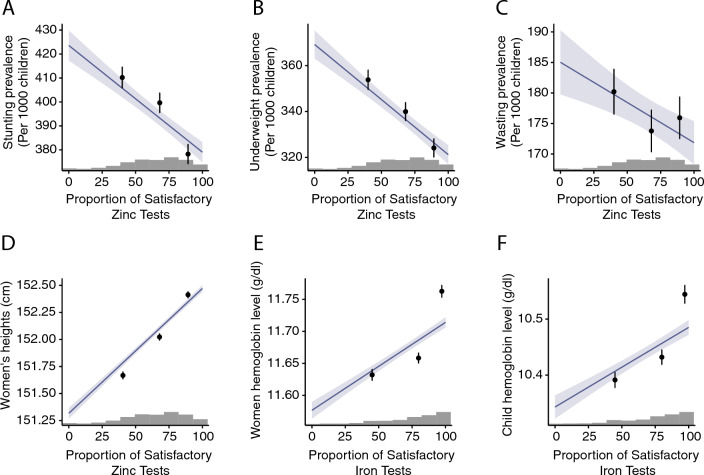
Response curves: Soil nutrient availability and health outcomes. Plots show the predicted response in health outcomes (vertical axis) to changes in soil nutrient availability. Purple lines show results from the linear models (see Eq. (1), "Methods" Section) with shaded regions showing 95% CI. The dot and whiskers show the model predictions at terciles of soil nutrient availability (Eq. (2), "Methods" Section) and associated 95% CI. (**A**), (**B**) and (**C**) show the association between soil zinc and children’s stunting, underweight, and wasting, respectively. (**D**) shows the relationship between soil zinc and women’s height. (**E**) and (**F**) show the response of women’s and children’s hemoglobin levels to soil iron, respectively. Companion histograms display the distributions of soil zinc and soil iron values that are assigned to children and women.

Our primary estimates show the existence of a statistically strong relationship between soil zinc availability and nutritional outcomes of children and adult women in India (Table 1, Fig. 2A–D). In particular, regressions including only minimal controls and region fixed effects demonstrate that districts with an increased proportion of satisfactory soil zinc samples have significantly lower rates of child stunting and underweight. A one standard deviation increase in satisfactory soil zinc (equivalent to a 24.3% increase in the proportion of satisfactory zinc tests) is associated with a reduction in stunting by 10.8 per 1000 children (95% CI [$-16.8, -4.86$]) and with a reduction in underweight by 11.7 per 1000 children (95% CI [$-18.7, -4.66$]). Soil zinc availability is also associated with an increase in the height of women. A one standard deviation increase in satisfactory soil zinc is associated with a 0.29 cm increase in women’s heights (95% CI [0.15, 0.43]). District-level soil zinc availability does not, however, appear to be correlated with child wasting. This is in line with nutrition studies showing a strong relationship between child zinc status and linear growth but only a tenuous relationship between child zinc status and weight gain^29,31^.

**Table 1:** Relationship between soil zinc availability and health outcomes among children and adult women.

	Stunting	Underweight	Wasting	Women’s height
	(1)	(2)	(3)	(4)
District-level soil Zn availability (% Satisfactory)	$$-0.445{***}$$	$$-0.480{***}$$	− 0.132	$$0.012{***}$$
	(0.125)	(0.147)	(0.105)	(0.003)
Individual controls	Yes	Yes	Yes	Yes
Region fixed effects	Yes	Yes	Yes	Yes
N	226,195	226,195	226,195	664,849
$\hbox {R}^{2}$	0.02	0.03	0.01	0.02

Each cell in the table shows estimates from a separate OLS regression using Eq. (1) (see "Methods" Section). All specifications include controls for individual characteristics (mother’s religion, ethnicity, age at childbirth, age at start of first marriage, child’s gender, birth order, and month of birth, whether the child lives in an urban or rural location in columns (1–3); age, religion, ethnicity, urban/rural, and month of birth for column (4)). Outcome variables in columns (1–3) are children’s anthropometric measures defined as binary variables taking value 1000 if a given child was stunted, wasted, or underweight, and 0 otherwise. The outcome in Column (4) is women’s height measured in centimeters (cm). The coefficient on district-level soil Zn shows the change in prevalence of the outcome (per ’000 children) in columns (1–3) and the change in height (cm) (column 4) for a 1% increase in the number of satisfactory soil Zn tests in a district. Values in parentheses show the standard errors clustered at the district level. Coefficient significance at 1%, 5% and 10% are indicated by $${***}$$, $${**}$$ and $${*}$$, respectively.

The second set of results in our analysis suggests a strong relationship between soil iron availability and hemoglobin levels among children and women (Table 2, Fig. 2E,F). A one standard deviation increase in satisfactory iron (equivalent to a 26.8% increase in the proportion of satisfactory iron tests) is associated with a 0.038 g/dL increase in hemoglobin for children (95% CI [0.0011, 0.0751]) and a 0.037 g/dL increase in hemoglobin for women (95% CI [0.0024, 0.0713]). Our findings on the association between soil iron and hemoglobin are particularly important from a public health perspective. India has one of the highest prevalences of anemia: 53.1% of women and 58.5% of children under 5 are anemic^35^. Our results suggest that soil mineral availability is a potential channel that could be leveraged to mitigate the prevalence of anemia across a large section of the population.

**Table 2:** Relationship between soil iron availability and hemoglobin levels among children and adult women.

	Children’s hemoglobin	Women’s hemoglobin
	(1)	(2)
District-level soil Fe availability (% Satisfactory)	0.001$$^{**}$$	0.001$$^{**}$$
	(0.001)	(0.001)
Individual controls	Yes	Yes
Region fixed effects	Yes	Yes
N	198,906	657,770
$\hbox {R}^{2}$	0.03	0.01

Each cell in the table shows estimates from a separate OLS regression using Eq. (1) (see "Methods" Section). Column (1) includes controls for child characteristics (mother’s religion, ethnicity, age at childbirth, age at start of first marriage, child’s gender, birth order, and month of birth, whether the child lives in an urban or rural location). Column (2) includes controls for women’s characteristics (age, religion, ethnicity, urban/rural, and month of birth). The outcome variable in both columns is hemoglobin levels measured in *g*/*dL*. The coefficient on district-level soil Fe shows the change in hemoglobin levels for a 1% increase in the number of satisfactory soil Fe tests in a district. Values in parentheses show the standard errors clustered at the district level. Coefficient significance at 1%, 5% and 10% are indicated by ***, ** and *, respectively.

### Robustness tests

#### Alternate regression specifications

The estimates we obtain from our linear model remain consistent when we use a binned model to account for differential impacts across different levels of soil mineral availability (Extended Data Tables 1 and 2). We also evaluate the robustness of our models to the inclusion of additional controls. These include adding other district-level soil micro- and macro-nutrient tests (e.g., percentage satisfactory samples for nitrogen, phosphorus, and calicium; see full list in Table 4), as well as controls for fertilizer use, because of concerns about omitted variable bias. The inclusion of these controls provides some assurance that high soil zinc or iron availability is not simply capturing high soil fertility more generally or reflecting greater fertilizer application and, therefore, wealth. We find that in models controlling for fertilizer use, all relationships significant in the original models remain significant. In the models controlling for other soil micro- and macro-nutrients, the relationship between soil zinc and stunting remains significant, but the relationships between other micronutrients and health outcomes become insignificant (Extended Data Tables S3–S8).

#### Causal bounds test

Next, we undertake Oster’s (see "Methods" Section)^36^ statistical sensitivity test to explore whether unobservable characteristics could manufacture the soil-health relationships we find in Tables 1 and 2. This test necessitates an assumed value for the parameter $R^{max}$, which reflects the explanatory power of unobservables as explained in the "Methods" Section. Under our first assumption—a threshold value based on results from a variety of other randomized studies—we estimate that the biasing power of unobserved covariates would have to be stronger than that of observed controls ($\delta _{1} > 1$) in order to spuriously manufacture the observed relationship between soil characteristics and all nutrition outcomes except for women’s height (Table 3, $\delta _{1}$). Under our second assumption—$R^{max}$ values based on adding district-level fixed effects to the regressions in Tables 1 and 2—we estimate that $\delta _{2} > 1$ only for the relationship between soil zinc and child stunting (Table 3, $\delta _{2}$). For this reason, we consider the relationship between soil zinc and child stunting most likely to be causal (as opposed to associational or driven by unobserved spatial factors) and move on to examine heterogeneity in only this relationship.

**Table 3:** Selection on unobservables necessary to manufacture soil-nutrition associations.

Outcome	$\delta _{1}$	$\delta _{2}$
Stunting incidence (per ’000)	4.642261	1.553850
Underweight incidence (per ’000)	2.686811	0.637739
Wasting incidence (per ’000)	1.241936	0.125159
Women’s height (cm)	0.821860	0.254413
Children’s hemoglobin (g/dL)	1.344673	0.138296
Women’s hemoglobin (g/dL)	4.219345	0.287656

$\delta _{1}$ and $\delta _{2}$ provide the ratio of selection on unobservables—selection on observables that would be necessary to manufacture the soil-nutrition associations observed in Tables 1 and 2 in the absence of any true, causal relationship, according to Oster’s (2019) statistical sensitivity test^36^. $\delta _{1}$ is calculated based on an $R^{max}$ threshold of 1.3 multiplied by the $R^{2}$ from Table 1. $\delta _{2}$ is calculated under the assumption that $R^{max}$ is the $R^{2}$ obtained by adding district fixed effects to the regression models in Eq. (1).

### Heterogeneity in the soil zinc-stunting relationship

**Figure 3:**
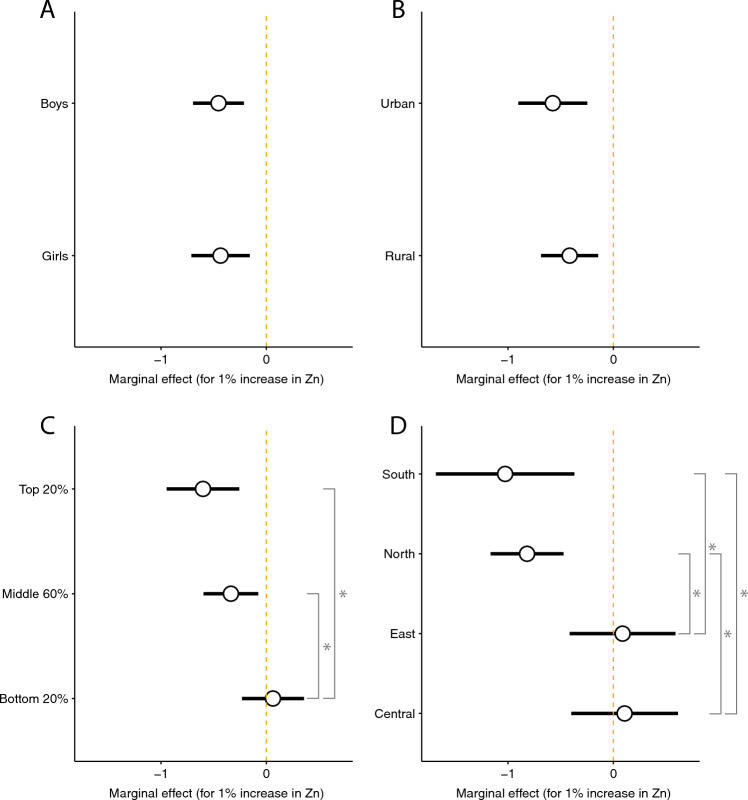
Heterogeneous effect of a 1% increase in soil zinc availability on child stunting by (**A**) gender, (**B**) urban or rural location, (**C**) household asset wealth index (top and bottom 20% and middle 60%), and (**D**) region. Circles provide point estimates and whiskers represent 95% confidence intervals. Stars represent significantly different marginal effects at the 0.05% level.

Using Eq. (3) (see "Methods" Section), we examined heterogeneity by gender, urban and rural location, household wealth, and region. We see no statistically significant difference in the strength of the soil zinc-stunting relationship for boys vs. girls, or for children living in urban vs. rural locations (Fig. 3A,B). However, the marginal effect of soil zinc availability on child stunting does differ significantly across household wealth categories (Fig. 3C): the relationship is strongest for the wealthiest households (wealth in the top 20%) and weakest for the poorest households (wealth in the bottom 20%). The marginal effect is also stronger for children living in the north and south regions of India than for children living in the east and central regions (Fig. 3C,D). Since households in the north and south regions are more likely to be wealthy than households in the central and east regions (Fig. S1), it may be that differential wealth levels drive the strong marginal effects in the north and south. The north and south regions do not have markedly different stunting rates or satisfactory soil zinc rates than the other regions (Figs. S2, S3). Limitations of sample size imply we are likely underpowered to detect significant differences when examining heterogeneity by household wealth level across regions (Fig. S4).

## Discussion

This study suggests that soil mineral availability may have a causal impact on the nutritional status of women and children in India. District-level soil zinc availability predicts child stunting, child underweight, and women’s height. District-level soil iron availability predicts both children’s and women’s hemoglobin levels. These initial relationships were examined via linear and binned models each with region fixed effects, thus identifying on district-to-district soil variation within (not across) any given region. When state fixed effects were instead added to the models, identifying on within-state, district-level variation in soils only, and/or when controls for other soil characteristics were added to models, the relationships between soil zinc/iron availability and health outcomes became weaker and generally less precise. Yet this is unsurprising since state fixed effects reduce identifying variation in soil mineral availability, reducing the power of our hypothesis test. Two variations of Oster’s (2019)^36^ statistical sensitivity test suggest that the relationship between soil zinc availability and child stunting is unlikely to be spurious. One of those variations suggests that the relationship between soil iron availability and children’s and women’s hemoglobin is also unlikely to be spurious.

This is the first paper to examine large-scale relationships between soil mineral availability and human nutritional status in India, though scientists have speculated about the relationship between soil zinc and human zinc status in India for over a decade^8,26,37^. Our findings are consistent with previous work that has found soil mineral (zinc or selenium) availability predicts human nutritional status in Malawi, Ethiopia, and Nepal^10,12–14,17^. Our results also align with a study of 15 farming families in Madhya Pradesh, which found that soil zinc availability predicted rice zinc concentration, which in turn predicted family member zinc status^18^. Ours is the first paper we know of to find a linkage between soil iron availability and human (maternal and child) hemoglobin concentration, and therefore risk of anemia. However, we find that the most robust soil-health relationship in India is between soil zinc availability and child stunting, similar to findings from Nepal^17^. More generally, our findings contribute to the literature on the effect of environmental factors like air pollution, sanitation, or prevalence of aflatoxins on child stunting^38–40^. The context of India is especially important for research on the determinants of micronutrient malnutrition, as India contains over one-third of the world’s malnourished children^41^.

Interestingly, the relationship between soil zinc availability and women’s height was not as statistically robust as between soil zinc availability and child stunting. This, too, mirrors results found in Nepal^17^. One possible explanation is that a non-negligible number of adult women have moved across districts at some point in their lives, obscuring the effect of their childhood soil conditions. It is also possible that constraints to height were more numerous for older generations, making the effect of dietary zinc intake less impactful for childhood growth (and hence adult height). More optimistically, some adolescents may experience catch-up growth that mitigates the height impacts of reduced childhood zinc intake^42,43^. If catch-up growth occurs, it is important to ask whether positive adolescent growing conditions similarly mitigate other long-term cognitive or non-cognitive effects of childhood malnutrition.

It is possible that multiple mechanisms underlie the link between soil zinc/iron availability and human height/anemia. Experimental studies show that soil zinc deficiency impedes both the yields of cereals in India, and the zinc concentration in the edible portion of these crops^44–47^. Soil zinc availability can therefore impact child linear growth directly by influencing local dietary zinc intake. It may also impact child growth indirectly by influencing local yields, income/poverty, and more general child health. In Nepal, both mechanisms are likely at play^17^. Similarly, soil iron deficiency may impede crop iron concentration and local dietary iron intake, driving anemia. Soil iron availability may also be correlated with local groundwater iron concentration, which is protective against human anemia in Bangladesh^48–51^, and therefore possibly in India. In this paper, we do not attempt to validate or disentangle these various mechanisms.

In heterogeneity analysis, we find that the strongest association between soil zinc and child stunting is in the wealthiest households. This was somewhat unexpected given the past work that has linked poverty and soil quality^52^. However, poorer households may face a great variety of constraints to childhood growth, just as older generations in India or Nepal may have, which would limit the importance of a single factor such as soil zinc availability (and, through that, dietary intake) on child stunting.

This study has implications for understanding the appropriateness of micronutrient-enriched fertilizer application in India. Foliar and soil application of minerals like zinc, iron, boron, and sulphur to wheat and rice has been shown to increase the plant concentrations of these minerals, as well as yields, both within and outside of India^8,44–47,53–59^. In India specifically, application of zinc to crops grown on zinc-deficient soils increases yields of rice, wheat, maize, and oats by over 75% more than application of only nitrogen, phosphorus, and potassium fertilizer^60^. Application of zinc-enriched fertilizers can enhance soil zinc for 3 to 4 years after application, which means that it could be an effective long-term intervention, requiring less short-term maintenance than other solutions^61^. This study suggests that agronomic fortification may be a method by which to reduce micronutrient deficiency in India, although more work is needed to also consider the costs of such an intervention.

One limitation of our study is the fact that every person in a district was assigned to the same soil mineral availability. In India, internal agricultural trade has substantial barriers such as a lack of adequate transport infrastructure. Agricultural trade is closely regulated by both central and state government policies and is often constrained by state and district-level tariffs^62^. As a result, past studies have found that district-level factors are most closely associated with differences in consumption of staple cereals, pulses, and dietary diversity^63,64^. Yet, cereals, legumes, and other food goods are traded across districts, particularly within states. This means that for most families, district-level soil zinc or soil iron availability is an errored measure of true dietary exposure to multi-district soil zinc or iron. This measurement error should cause us to underestimate the true relationship between dietary exposure to soil zinc or iron availability and human nutritional status. That is, the causal soil-health relationships may be larger than the associations estimated in this paper.

While sample-level geocoded data on soil quality are available from the Soil Health Card scheme, they were not used because district-level soil nutrient exposure seems an appropriate treatment variable, and because of a mismatch in test counts between the sample-level and district-level data. Several districts with the lowest sample-level test counts had much higher district-level test counts, which suggests that the district-level data we use are more reliable than the publicly available sample-level data for the purposes of this study (Fig. S5A). It should be noted that the majority of districts had lower test counts at the district level than the sample level, suggesting that the district-level data does not incorporate all of the samples that have been taken in a given district (Fig. S5A). This is possibly because the district-level aggregates discard bad-quality samples.

## Conclusions

The growing availability of georeferenced data on both health and nutrition outcomes and soil nutrients offers new opportunities to understand the role of edaphic factors in food security and health. Here we utilize data from over 27 million soil tests and health surveys of over one million individuals in India to assess the importance of soil zinc and iron availability for two important health outcomes: measures of physical growth and hemoglobin levels. A key challenge in this endeavor is the cross-sectional nature of the data, given that soil properties change very slowly over time. By comparing our base results with results from models that incorporate further soil (nutrients and fertilizer) controls and finer-grained geographic fixed effects, we identified several plausible effects of soil conditions on health outcomes, with some more robust to model specification and unobserved variables than others. The most robust finding is that low soil zinc availability is associated with childhood stunting. This effect appears to be strongest in wealthier households, perhaps reflecting the fact that children from poorer households face many more health constraints beyond zinc status that contribute to stunting. Overall, our results suggest that the potential benefits of agronomic zinc interventions deserve more consideration, in India specifically and perhaps more generally.

## Methods

### Data

#### Soil data

Soil data used in this study came from India’s Soil Health Card scheme. This program tests soil samples from farmers across India for soil quality characteristics such as micronutrients, macronutrients, and soil pH in order to give farmers information about the quality of their soil and inform their choices for soil quality management actions such as fertilizer application^65^. These data are publicly available at the district level, aggregated to the proportion of “satisfactory” and “unsatisfactory” tests per district for each soil characteristic, where the Indian government sets “satisfactory” and “unsatisfactory” standards (Fig. 1A,B)^66^. For this study, data from the 2017 to 2019 testing period were used, which comprised over 27 million tests^67^.

Importantly, the standards for “satisfactory” and “unsatisfactory” designations reflect nutrients needed for crop health, not necessarily for human health. Crops have been shown to take up zinc in soil beyond necessary zinc for plant growth^68^, meaning that crop-based standards likely underestimate the true benefit of crops growing on soils extremely high in zinc.

District-level soil quality aggregates are appropriate for this study for several reasons. In some areas of India, rural households rely on their own production for about half of their consumption^64^. As a result, our measure of district-level soil mineral availability likely captures the scale of soil mineral exposure most relevant to local nutritional status. Furthermore, from a policy perspective, districts and states in India are the primary administrative level at which health and agricultural interventions are tested and implemented^69,70^.Indian agricultural marketing laws also restrict farmers to selling their produce to intermediaries within their own state, with the majority of sales occurring with intermediaries who are located in close spatial proximity (usually within the same district) to farmers^71^. Therefore, the use of a district-level measure corresponds with the spatial scale at which potential agronomic fortification interventions could be introduced.

To explore the variation in district-level aggregates, sample-level data from the Soil Health Card in the state of Bihar were compared to district-level data. This comparison indicated that there is an agreement between sample-level and district-level information (Fig. S5). However, sample-level data were not used for the study because, in many districts, the district aggregates are based on more individual sample tests than we have public access to (Fig. S5A). For these districts, the district-level aggregates are, therefore, a better measure of soil quality than our own aggregates of the selected sample-level data. Secondarily, even in districts where all individual samples are available or where district-level aggregates were based on fewer tests than those available at the sample level, it is possible that district-level aggregates were made based on greater information than we have. For instance, data from poorly run labs may have been discarded, or the definition of “satisfactory” may have been tweaked according to variation in the test protocols across labs. Thus, district soil data aggregates were used in this study instead of individual soil tests.

#### Health data

Data on children’s and women’s health, as well as data for study controls, were drawn from India’s 2015-16 National Family Health Survey (NFHS) data, part of the Demographic and Health Survey series^72^. The NFHS data are available at the individual level for approximately 250,000 children and approximately 700,000 women in India (Fig. 1C,D). The health metrics evaluated for children were stunting (low height for age) (mean = 36.9%, s.d. = 48.2%), wasting (low weight for height) (mean = 19.6%, s.d. = 39.7%), underweight (low weight for age) (mean = 33.2%, s.d. = 47.1%), and hemoglobin levels (grams/deciliter) (mean = 10.62, s.d. = 1.54). The health metrics evaluated for women were women’s heights (cm) (mean = 152.0, s.d. = 6.14) and hemoglobin levels (mean = 11.74, s.d. = 1.66). We drop observations for which the relevant health metrics data was not collected in the survey or the control variables were missing. Children were determined to be stunted, wasted, or underweight based on whether their z-score for height-for-age, weight-for-height or weight-for-age, respectively (based on WHO standards), was below $-2$ (ICF 2018). All analyses were performed in accordance with relevant guidelines and regulations. Institutional approval of experimental protocols and informed consent from the women and children surveyed were not necessary for this study, because all data analyzed were anonymized secondary data.

#### Additional data

Fertilizer data were drawn from the International Crops Research Institute for the Semi-Arid Tropics’ 2015-2017 datasets on total fertilizer use in kilograms per net hectare cropped^73^. Data were averaged across these years by district. Since the data cover only 20 states in India, a dummy variable for districts without fertilizer information was entered into regression models when the fertilizer use variable was entered.

Soil and health data were joined by assigning individuals to soil quality metrics based on district of residence. A person’s district was determined based on the latitude and longitude of the person’s cluster in the NFHS survey (Fig. 1D)—individuals are assigned to clusters, which approximate villages with a spatial jitter of 0–10 km^74^, with the constraint that clusters are not jittered outside of district boundaries. All individuals in a cluster, therefore, had the same latitude/longitude coordinates associated with them, and all individuals in a district were assigned the same soil quality metrics.

### Empirical strategy

Linear and binned regressions were performed, modeling individual (children's or women’s) health outcomes as a function of the proportion of satisfactory soil mineral tests in a district. The outcome variables of child stunting, wasting, and underweight were defined as binary variables taking the value 1000 (to ascertain soil quality’s association with stunting, wasting, and underweight prevalence per 1000 children) if a given child was stunted, wasted, or underweight, and 0 otherwise. Hemoglobin levels (grams/deciliter) and women’s heights (cm) are continuous outcome variables.

These regressions pick up associations only (not causal relationships) because soil zinc and iron availability are not exogenously/experimentally determined. For instance, variation in soil zinc availability is determined by soil zinc concentrations, pH, calcite and organic matter, and concentrations of other micronutrients and macronutrients like sodium, calcium, and magnesium, as well as bicarbonate and phosphate^75^. Soil zinc concentration itself is generally driven by geologic factors^75^. It is also well known that application of nitrogen, phosphorus, and potassium fertilizers reduces the uptake of soil zinc. Availability of soil zinc is thus affected by fertilizer type, likely increasing with application of zinc-enriched fertilizers (which have been used in India since the 1960s) and decreasing with application of fertilizers not enriched with zinc^26^.

**Table 4:** List of control variables used in regression models.

Model	List of control variables
Minimal controls (Children)	Mother’s religion, ethnicity, age at childbirth, age at start of first marriage child’s gender, birth order, and month of birth, whether the child lives in an urban or rural location
Minimal controls (Women)	Age, religion, ethnicity, month of birth, whether the woman lives in an urban or rural location
Soil controls (zinc models)	Percentage of samples in a district that were satisfactory for iron, copper, manganese, boron, sulfur, nitrogen, phosphorus, potassium, and organic carbon
Soil controls (iron models)	Percentage of samples in a district that were satisfactory for iron, copper, manganese, boron, sulfur, nitrogen, phosphorus, potassium, and organic carbon
Fertilizer controls	Kilograms of fertilizer use per hectare of net cropped area in district (average of 2015 to 2017)

Because soil zinc and iron availability are not exogenously determined, we include a variety of controls in our regressions to account for potential confounding factors and, therefore, move the regression coefficients closer to the causal relationships of interest. Control variables are summarized in Table 4. We chose controls that were not likely to be driven by soil quality, so as not to introduce bias due to “bad controls”^76^. Additionally, we include either state or region fixed effects in all models. This allows us to compare individuals exposed to differing levels of soil nutrients across districts within the same region or state, thereby cleansing any correlation between soil nutrients and state/regional characteristics such as infrastructure, governance, crop procurement prices, etc. Regions are defined as aggregations of states geographically close to one another (Table S9). States were grouped into regions on the basis of similarities in cropping patterns, dietary tastes, and shared cultural, linguistic, and kinship structures. The classification follows grouping defined in the sociological literature and used in previous studies examining regional disparities in demographic and health outcomes across India^77–79^.

Linear and binned models were evaluated using Eqs. (1) and (2), respectively, for model formulation. Models of heterogeneity were evaluated using Eq. (3).

1 $$\begin{aligned} Y_{i,d}&= \beta ^{0} + \beta ^{1}Z_{i,d} + \beta ^{2}X_{i,d} + c_{r} + \varepsilon _{i,d} \end{aligned}$$

2 $$\begin{aligned} Y_{i,d}&= \beta ^{0} + \beta ^{1}Z^{high}_{d} + \beta ^{2}Z^{medium}_{d} + \beta ^{3}X_{i,d} + c_{r} + \varepsilon _{i,d} \end{aligned}$$

3 $$\begin{aligned} Y_{i,d}&=\beta ^{0} + \beta ^{1}Z_{i,d}*V_{i,d} + \beta ^{2}Z_{i,d} + \beta ^{3}V_{i, d} + \beta ^{4}X_{i,d} + c_{r} + \varepsilon _{i,d} \end{aligned}$$

The outcome for individual *i* living in district *d* is given by $Y_{i,d}$. $Z_{d}$ denotes the treatment variable of interest for linear models, the percent of satisfactory soil zinc or soil iron tests in district *d*. In the binned models, soil zinc or iron levels are binned into terciles, with the lowest bin as the reference quantile, $Z^{high}_{d}$ indicating that district *d* falls in the highest quartile, and $Z^{medium}_{d}$ indicating that district *d* is in the medium quartile. $V_{d}$ represents the heterogeneous effects variable being investigated, which is incorporated through an interaction with the treatment variable of interest. Controls for individual characteristics are given by $X_{i,d}$—these controls are listed in Table 4 as “minimal controls”, and we include them in all linear and binned specifications. We also include fixed effects for state-aggregate regions ($c_{r}$) in all specifications; these regions are defined in Table S9. In subsequent robustness checks, we include controls for district-level soil characteristics and fertilizer application, again defined in Table 4, and include state-level rather than region-level fixed effects. Standard errors are clustered at the district level.

#### Oster’s causal bounds method

We evaluate the robustness of the observed relationships between soil mineral availability and health outcomes in Model 1, and consider whether unobserved, relevant covariates may spuriously drive the observed soil-health associations. To do so, we use a statistical sensitivity test that gauges how important unobserved covariates would have to be to spuriously manufacture our observed soil-health relationships if no true, causal relationship existed^36^. This method, proposed by Oster (2019), quantifies the necessary importance of unobserved covariates vis-à-vis the importance of observed covariates.

Oster’s method requires an assumption regarding $R^{max}$, the hypothetical value of model $R^{2}$ if all unobserved covariates were entered into the model alongside the observed covariates ($X_{i,d}$) that are already included in Eq. (1). We run this sensitivity test under two different $R^{max}$ assumptions. First, we follow Oster’s rule of thumb by assuming that for each outcome, $R^{max}$ would be 1.3 times higher than the $R^{2}$ observed in Eq. (1). Second, we assume that for each outcome, $R^{max}$ would be equivalent to the $R^{2}$ obtained by adding district-level fixed effects to Eq. (1). Because the first assumption provides a higher $R^{max}$ than the second, the first assumption is more conservative, i.e., will be more likely to suggest that soil-health relationships are spuriously driven by unobserved variables. Both methods were described by Oster (2019) as useful ways to approximate $R^{max}$.

### Supplementary Information

Supplementary Information.

## Data Availability

The datasets generated during the current study are available from the corresponding author on reasonable request. For datasets including the Bihar villages shapefile, data are only available to affiliates of Stanford University or on proof of license to use the dataset. For datasets including data from the NFHS, data are available on proof of registration with the NFHS. Code and publicly available data (all data other than the Bihar villages shapefile and the NFHS datasets) are available through Zenodo under the 10.5281/zenodo.7811067. The README file in the Zenodo contains information to access all datasets used in the paper.
