# Supplementary material for: Soil micronutrients linked to human health in India
Source: Sci Rep. 2023 Aug 21;13:13591. doi: 10.1038/s41598-023-39084-8 (PMC10442378; doi:10.1038/s41598-023-39084-8)
Supplement: Supplementary file 1 — Supplementary Information. [file 41598_2023_39084_MOESM1_ESM.pdf]

Soil Micronutrients Linked to Human Health in India

Claire M. Morton*[1], Hemant Pullabhotla[2], Leah Bevis[3], David B. Lobell[4]

[1]Mathematical and Computational Science Program, Stanford University, Stanford, USA
[2]Department of Economics, Deakin University, Burwood, Australia
[3]Department of Agricultural, Environmental & Development Economics, Ohio State University, Columbus, USA
[4]Department of Earth System Science and Center on Food Security and the Environment, Stanford University, Stanford, USA

Supplementary Figures

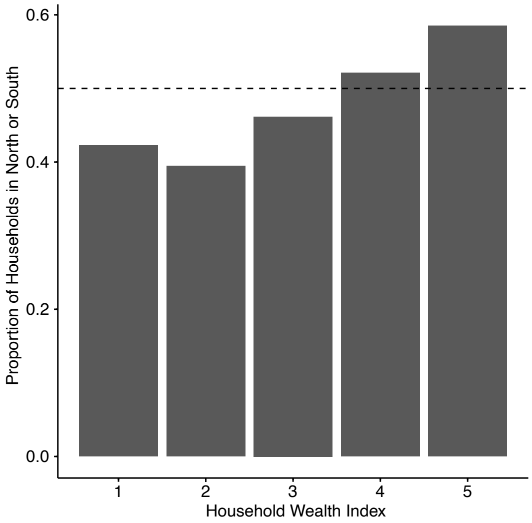

Figure S1. Proportion of sample households within each asset wealth index quintile that reside in the North or South. Bars show the proportion of North and South households within each wealth index quantile. The horizontal dashed line indicates 0.5. North and South households comprise a higher proportion (above 0.5) of the top two wealth index quintiles. The wealth index is a measure provided by the NFHS-IV data based on asset ownership of households.

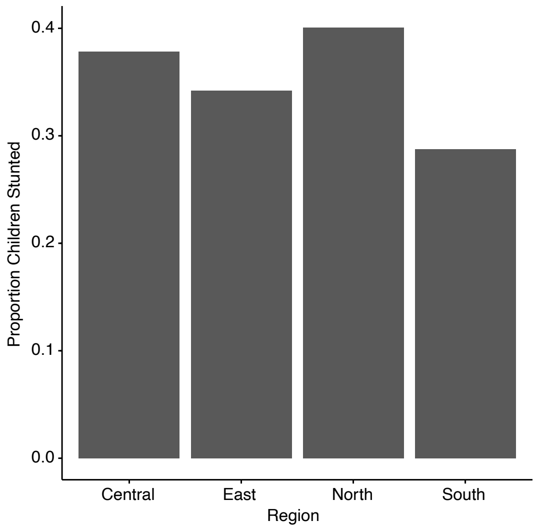

Figure S2. Proportion of stunted children under-5 within each region. Children are classified as stunted if their height-for-age z-score is below -2.

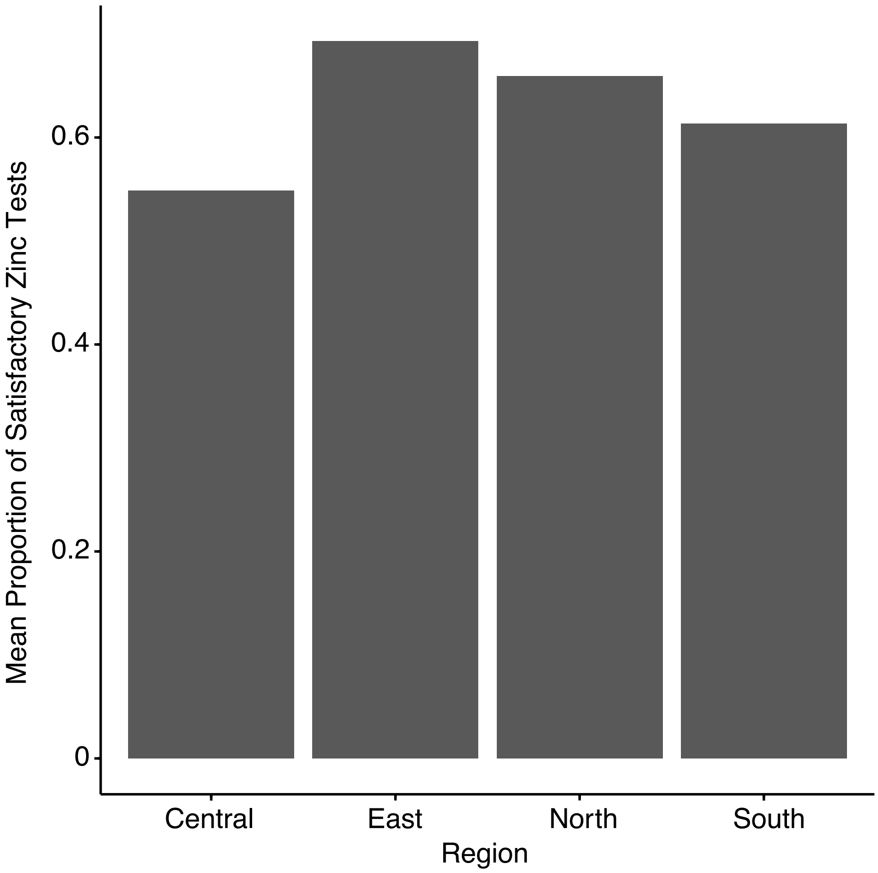

Figure S3. Mean proportion of satisfactory zinc tests, by region. Average of district proportions of satisfactory zinc soil tests.

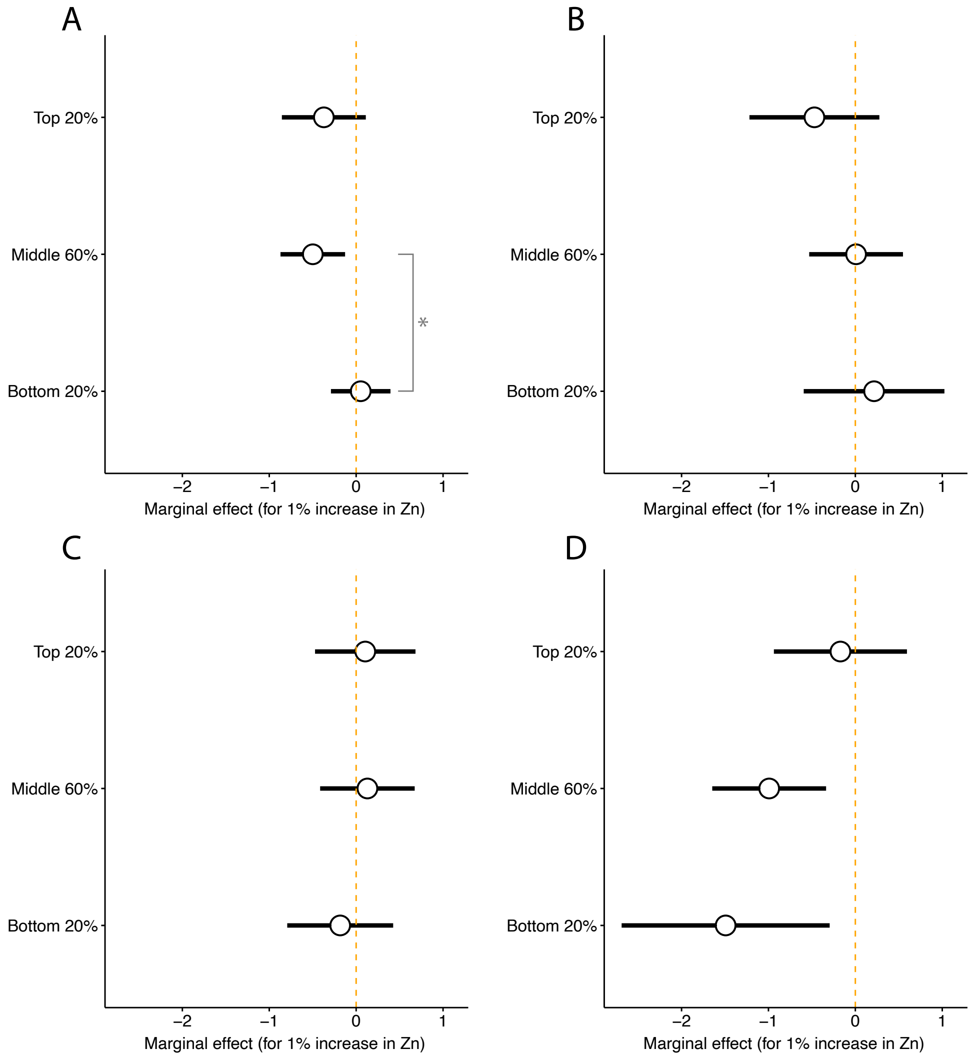

Figure S4. Heterogeneous effect of zinc on stunting in children for 1% increase in zinc by (A) north, (B) central, (C) east, and (D) south regions. Circles show the point estimates and the whiskers represent 95% confidence intervals. Stars represent significantly different marginal effects at the .05 level.

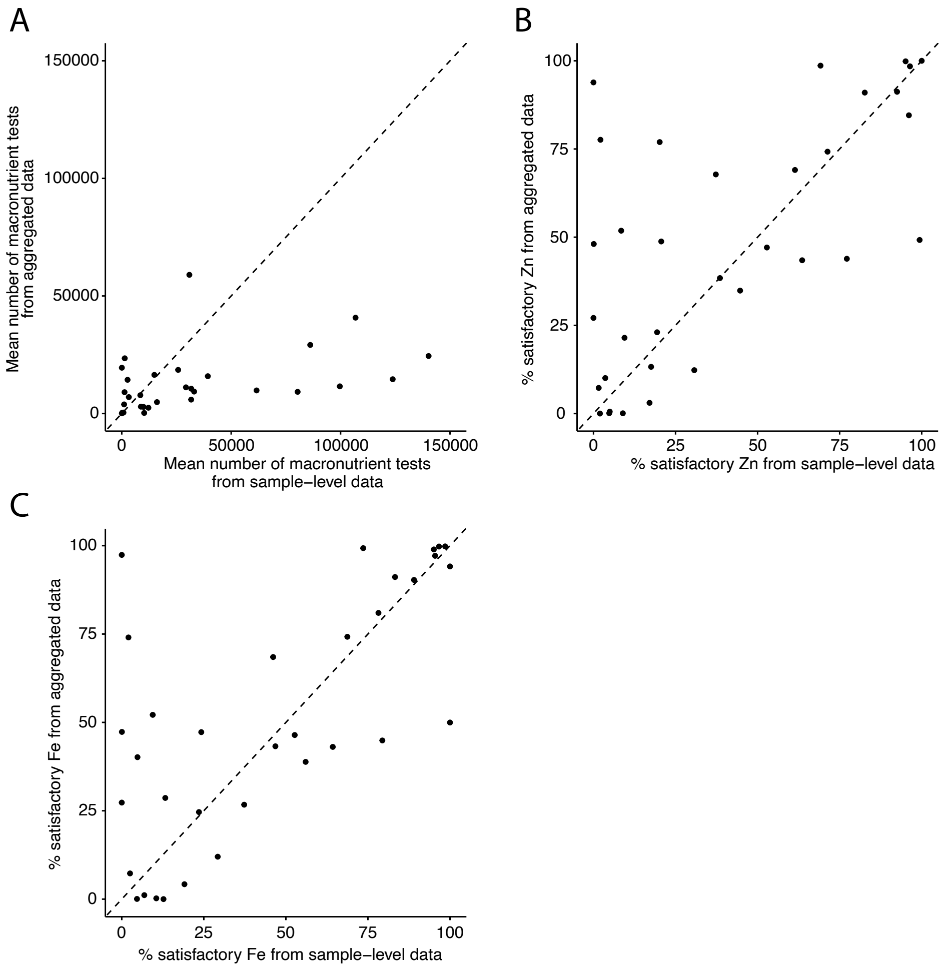

Figure S5. Data validation for national soil data comparing sample-level data from Bihar to district-aggregated data. (A) compares test counts for districts in Bihar, (B) compares the proportion of satisfactory zinc samples for districts, and (C) compares the proportion of satisfactory iron samples for districts.

Supplementary Tables

Extended Data Table 1 — Soil zinc and health outcomes: binned model results

	Stunting	Underweight	Wasting	Women's Height
	(1)	(2)	(3)	(4)
Zn middle tercile	−10.549	−13.856	−6.434	0.355**
	(6.982)	(8.887)	(6.477)	(0.154)
Zn high tercile	−31.946***	−29.705***	−4.276	0.746***
	(7.859)	(9.756)	(6.761)	(0.161)
Individual controls	Yes	Yes	Yes	Yes
Region fixed effects	Yes	Yes	Yes	Yes
N	226,195	226,195	226,195	664,849
R^2	0.02	0.03	0.01	0.02

Notes: Each cell in the table shows estimates from a separate OLS regression using Equation (2) (see Methods). Columns (1) to (3) include controls for children's individual characteristics (mother's religion, ethnicity, age at child-birth, age at start of first marriage, child's gender, birth order, and month of birth, whether the child lives in an urban or rural location). Column (4) includes controls for women's age, religion, ethnicity, urban/rural, and month of birth. Outcome variables in columns (1) to (3) are children's anthropometric measures defined as binary variables taking value 1000 if a given child was stunted, wasted, or underweight, and 0 otherwise. The outcome in Column (4) is women's height measured in centimeters (cm). The soil variables of interest are district-level satisfactory zinc tests binned into terciles, with the lowest bin as the reference category, Zn high tercile indicating that the district falls in the highest tercile, and Zn middle tercile indicating that the district falls in the second tercile. Values in parentheses show the standard errors clustered at the district level. Coefficient significance at 1%, 5% and 10% are indicated by ***, ** and *, respectively.

Extended Data Table 2 — Soil iron and hemoglobin: binned model results

	Children's Hemoglobin	Women's Hemoglobin
	(1)	(2)
Fe middle tercile	0.041	0.026
	(0.050)	(0.043)
Fe high tercile	0.153***	0.131***
	(0.056)	(0.049)
Individual Controls	Yes	Yes
Region fixed effects	Yes	Yes
N	198 906	657 770
R-squared	0.03	0.01

Notes: Each cell in the table shows estimates from a separate OLS regression using Equation (2) (see Methods). Column (1) includes controls for children's individual characteristics (mother's religion, ethnicity, age at childbirth, age at start of first marriage, child's gender, birth order, and month of birth, whether the child lives in an urban or rural location). Column (2) includes controls for women's age, religion, ethnicity, urban/rural, and month of birth. The outcome variable in both columns is hemoglobin levels measured in g/dL. The soil variables of interest are district-level satisfactory iron tests binned into terciles, with the lowest bin as the reference category, Fe high tercile indicating that the district falls in the highest tercile, and Fe middle tercile indicating that the district falls in the second tercile. Values in parentheses show the standard errors clustered at the district level. Coefficient significance at 1%, 5% and 10% are indicated by ***, ** and *, respectively.

Extended Data Table 3 — Linear models with soil controls and region-level fixed effects

	Stunting	Underweight	Wasting	Women's Height	Children's Hemoglobin	Women's Hemoglobin
	(1)	(2)	(3)	(4)	(5)	(6)
Zn	−0.305*	−0.274	−0.128	0.003	0.001	0.001
	(0.181)	(0.252)	(0.167)	(0.003)	(0.001)	(0.001)
Fe	−0.281	−0.261	−0.099	0.002	0.002	0.000
	(0.173)	(0.231)	(0.158)	(0.003)	(0.001)	(0.001)
Individual Controls	Yes	Yes	Yes	Yes	Yes	Yes
Soil Controls	Yes	Yes	Yes	Yes	Yes	Yes
State FE	Yes	Yes	Yes	Yes	Yes	Yes
N	197 875	197 875	197 875	582 283	175 395	579 862
R-squared	0.03	0.03	0.01	0.03	0.05	0.02

Notes: Each cell in the table shows estimates from a separate OLS regression using Equation (1) (see Methods). Columns (1), (2), (3), and (5) include controls for children's individual characteristics (mother's religion, ethnicity, age at childbirth, age at start of first marriage, child's gender, birth order, and month of birth, whether the child lives in an urban or rural location). Columns (4) and (6) include controls for women's age, religion, ethnicity, urban/rural, and month of birth. Outcome variables in columns (1) to (3) are children's anthropometric measures defined as binary variables taking value 1000 if a given child was stunted, wasted, or underweight, and 0 otherwise. The outcome in Column (4) is women's height measured in centimeters (cm). The outcome variables columns (5) and (6) is hemoglobin levels measured in g/dL. The soil variables of interest are district-level percentage of soil tests with satisfactory soil nutrient levels. Values in parentheses show the standard errors clustered at the district level. Coefficient significance at 1%, 5% and 10% are indicated by ***, ** and *, respectively.

Extended Data Table 4 — Linear models with state-level fixed effects

	Stunting (1)	Underweight (2)	Wasting (3)	Women's Height (4)	Children's Hemoglobin (5)	Women's Hemoglobin (6)
Zn	−0.020	0.019	0.014	−0.002		
	(0.118)	(0.116)	(0.105)	(0.002)		
Fe					0.001**	0.000
					(0.001)	(0.001)
Individual Controls	Yes	Yes	Yes	Yes	Yes	Yes
State FE	Yes	Yes	Yes	Yes	Yes	Yes
N	226 735	226 735	226 735	667 347	199 387	660 263
R-squared	0.03	0.05	0.02	0.07	0.08	0.04

Notes: Each cell in the table shows estimates from a separate OLS regression using Equation (1) (see Methods). Columns (1), (2), (3), and (5) include controls for children's individual characteristics (mother's religion, ethnicity, age at childbirth, age at start of first marriage, child's gender, birth order, and month of birth, whether the child lives in an urban or rural location). Columns (4) and (6) include controls for women's age, religion, ethnicity, urban/rural, and month of birth. Outcome variables in columns (1) to (3) are children's anthropometric measures defined as binary variables taking value 1000 if a given child was stunted, wasted, or underweight, and 0 otherwise. The outcome in Column (4) is women's height measured in centimeters (cm). The outcome variables columns (5) and (6) is hemoglobin levels measured in g/dL. The soil variables of interest are district-level percentage of soil tests with satisfactory zinc in columns (1) - (4) and district-level percentage of soil tests with satisfactory iron in columns (5) - (6). Values in parentheses show the standard errors clustered at the district level. Coefficient significance at 1%, 5% and 10% are indicated by ***, ** and *, respectively.

Extended Data Table 5 — Linear models with fertilizer control and region-level fixed effects

	Stunting	Underweight	Wasting	Women's Height	Children's Hemoglobin	Women's Hemoglobin
	(1)	(2)	(3)	(4)	(5)	(6)
Zn	−0.466***	−0.533***	−0.163	0.012***		
	(0.119)	(0.128)	(0.101)	(0.003)		
Fe					0.001**	0.001**
					(0.001)	(0.001)
Individual Controls	Yes	Yes	Yes	Yes	Yes	Yes
Fertilizer	Yes	Yes	Yes	Yes	Yes	Yes
Region FE	Yes	Yes	Yes	Yes	Yes	Yes
N	226 195	226 195	226 195	664 849	198 906	657 770
R-squared	0.03	0.03	0.01	0.03	0.05	0.02

Notes: Each cell in the table shows estimates from a separate OLS regression using Equation (1) (see Methods). Columns (1), (2), (3), and (5) include controls for children's individual characteristics (mother's religion, ethnicity, age at childbirth, age at start of first marriage, child's gender, birth order, and month of birth, whether the child lives in an urban or rural location). Columns (4) and (6) include controls for women's age, religion, ethnicity, urban/rural, and month of birth. Outcome variables in columns (1) to (3) are children's anthropometric measures defined as binary variables taking value 1000 if a given child was stunted, wasted, or underweight, and 0 otherwise. The outcome in Column (4) is women's height measured in centimeters (cm). The outcome variables columns (5) and (6) is hemoglobin levels measured in g/dL. The soil variables of interest are district-level percentage of soil tests with satisfactory zinc in columns (1) - (4) and district-level percentage of soil tests with satisfactory iron in columns (5) - (6). Values in parentheses show the standard errors clustered at the district level. Coefficient significance at 1%, 5% and 10% are indicated by ***, ** and *, respectively.

Extended Data Table 6 — Binned models with soil controls and region-level fixed effects

	Stunting	Underweight	Wasting	Women's Height	Children's Hemoglobin	Women's Hemoglobin
	(1)	(2)	(3)	(4)	(5)	(6)
Zn terciles :						
2nd	−14.173*	−15.226	−4.403	0.369**	0.003	0.001
	(7.758)	(9.922)	(6.902)	(0.159)	(0.056)	(0.049)
3rd	−29.947***	−29.760**	−6.836	0.660***	0.017	0.037
	(10.000)	(12.798)	(8.623)	(0.189)	(0.065)	(0.058)
Fe terciles :						
2nd	1.981	0.111	0.014	−0.210	0.103*	0.042
	(8.600)	(10.226)	(7.041)	(0.173)	(0.056)	(0.048)
3rd	−15.088	−14.282	−2.728	−0.087	0.148*	0.128**
	(10.726)	(13.585)	(9.419)	(0.200)	(0.080)	(0.063)
Individual Controls	Yes	Yes	Yes	Yes	Yes	Yes
Soil Controls	Yes	Yes	Yes	Yes	Yes	Yes
Region FE	Yes	Yes	Yes	Yes	Yes	Yes
N	197 875	197 875	197 875	582 283	175 395	579 862
R-squared	0.03	0.03	0.01	0.03	0.05	0.02

Notes: Each cell in the table shows estimates from a separate OLS regression using Equation (2) (see Methods). Columns (1), (2), (3), and (5) include controls for children's individual characteristics (mother's religion, ethnicity, age at childbirth, age at start of first marriage, child's gender, birth order, and month of birth, whether the child lives in an urban or rural location). Columns (4) and (6) include controls for women's age, religion, ethnicity, urban/rural, and month of birth. Outcome variables in columns (1) to (3) are children's anthropometric measures defined as binary variables taking value 1000 if a given child was stunted, wasted, or underweight, and 0 otherwise. The outcome in Column (4) is women's height measured in centimeters (cm). The outcome variables columns (5) and (6) is hemoglobin levels measured in g/dL. The soil variables of interest are terciles of district-level percentage of soil tests with satisfactory zinc, and terciles of soil tests with satisfactory iron. The omitted category is the lowest (1st) tercile for each soil nutrient. The other soil nutrient control variables are also similarly binned into terciles. Values in parentheses show the standard errors clustered at the district level. Coefficient significance at 1%, 5% and 10% are indicated by ***, ** and *, respectively.

Extended Data Table 7 — Binned models with state-level fixed effects

	Stunting	Underweight	Wasting	Women's Height	Children's Hemoglobin	Women's Hemoglobin
	(1)	(2)	(3)	(4)	(5)	(6)
Zn terciles :						
2nd	−3.061	−1.156	−1.168	0.043		
	(6.128)	(6.985)	(6.086)	(0.084)		
3rd	−2.267	5.265	7.923	−0.104		
	(6.997)	(7.727)	(6.569)	(0.095)		
Fe terciles :						
2nd					0.063	0.024
					(0.045)	(0.039)
3rd					0.115**	0.059
					(0.047)	(0.043)
Individual Controls	Yes	Yes	Yes	Yes	Yes	Yes
State FE	Yes	Yes	Yes	Yes	Yes	Yes
N	226 735	226 735	226 735	667 347	199 387	660 263
R-squared	0.03	0.05	0.02	0.07	0.08	0.04

Notes: Each cell in the table shows estimates from a separate OLS regression using Equation (2) (see Methods). Columns (1), (2), (3), and (5) include controls for children's individual characteristics (mother's religion, ethnicity, age at childbirth, age at start of first marriage, child's gender, birth order, and month of birth, whether the child lives in an urban or rural location). Columns (4) and (6) include controls for women's age, religion, ethnicity, urban/rural, and month of birth. Outcome variables in columns (1) to (3) are children's anthropometric measures defined as binary variables taking value 1000 if a given child was stunted, wasted, or underweight, and 0 otherwise. The outcome in Column (4) is women's height measured in centimeters (cm). The outcome variables columns (5) and (6) is hemoglobin levels measured in g/dL. The soil variables of interest are terciles of district-level percentage of soil tests with satisfactory zinc, and terciles of soil tests with satisfactory iron. The omitted category is the lowest (1st) tercile for each soil nutrient. Values in parentheses show the standard errors clustered at the district level. Coefficient significance at 1%, 5% and 10% are indicated by ***, ** and *, respectively.

Extended Data Table 8 — Binned models with fertilizer controls and region-level fixed effects

	Stunting (1)	Underweight (2)	Wasting (3)	Women's Height (4)	Children's Hemoglobin (5)	Women's Hemoglobin (6)
Zn terciles :						
2nd	−9.515	−11.439	−5.103	0.342**		
	(6.720)	(7.686)	(6.015)	(0.147)		
3rd	−33.319***	−33.329***	−6.511	0.767***		
	(7.475)	(8.477)	(6.254)	(0.157)		
Fe terciles :						
2nd					0.046	0.039
					(0.047)	(0.040)
3rd					0.146***	0.111**
					(0.052)	(0.045)
Individual Controls	Yes	Yes	Yes	Yes	Yes	Yes
Fertilizer	Yes	Yes	Yes	Yes	Yes	Yes
Region FE	Yes	Yes	Yes	Yes	Yes	Yes
N	226 195	226 195	226 195	664 849	198 906	657 770
R-squared	0.03	0.03	0.01	0.03	0.05	0.02

Notes: Each cell in the table shows estimates from a separate OLS regression using Equation (2) (see Methods). Columns (1), (2), (3), and (5) include controls for children's individual characteristics (mother's religion, ethnicity, age at childbirth, age at start of first marriage, child's gender, birth order, and month of birth, whether the child lives in an urban or rural location). Columns (4) and (6) include controls for women's age, religion, ethnicity, urban/rural, and month of birth. Outcome variables in columns (1) to (3) are children's anthropometric measures defined as binary variables taking value 1000 if a given child was stunted, wasted, or underweight, and 0 otherwise. The outcome in Column (4) is women's height measured in centimeters (cm). The outcome variables columns (5) and (6) is hemoglobin levels measured in g/dL. The soil variables of interest are terciles of district-level percentage of soil tests with satisfactory zinc, and terciles of soil tests with satisfactory iron. The omitted category is the lowest (1st) tercile for each soil nutrient. Values in parentheses show the standard errors clustered at the district level. Coefficient significance at 1%, 5% and 10% are indicated by ***, ** and *, respectively.

Extended Data Table 9 — State and region assignments

State	Region
Andaman & Nicobar Islands	-
Andhra Pradesh	South
Arunachal Pradesh	East
Assam	East
Bihar	North
Chhatisgarh	Central
Dadra & Nagar Haveli	Central
Daman & Diu	Central
Goa	Central
Gujarat	Central
Haryana	North
Himachal Pradesh	North
Jammu & Kashmir	North
Jharkhand	East
Karnataka	South
Kerala	South
Ladakh	North
Madhya Pradesh	Central
Maharashtra	Central
Manipur	East
Meghalaya	East
Mizoram	East
Nagaland	East
NCT of Delhi	North
Orissa	East
Pondicherry	South
Punjab	North
Rajasthan	Central
Sikkim	East
Tamil Nadu	South
Telangana	South
Tripura	East
Uttar Pradesh	North
Uttarakhand	North
West Bengal	East
